# Bee Bread Ameliorates Vascular Inflammation and Impaired Vasorelaxation in Obesity-Induced Vascular Damage Rat Model: The Role of eNOS/NO/cGMP-Signaling Pathway

**DOI:** 10.3390/ijms22084225

**Published:** 2021-04-19

**Authors:** Zaidatul Akmal Othman, Zaida Zakaria, Joseph Bagi Suleiman, Victor Udo Nna, Aminah Che Romli, Wan Syaheedah Wan Ghazali, Mahaneem Mohamed

**Affiliations:** 1 Department of Physiology, School of Medical Sciences, Universiti Sains Malaysia, Kubang Kerian 16150, Kelantan, Malaysia; zaidaakmal@unisza.edu.my (Z.A.O.); zaida_zakaria@ymail.com (Z.Z.); aminahr@usm.my (A.C.R.); syaheeda@usm.my (W.S.W.G.); 2 Unit of Physiology, Universiti Sultan Zainal Abidin, Kuala Terengganu 20400, Terengganu, Malaysia; 3 Department of Science Laboratory Technology, Akanu Ibiam Federal Polytechnic, P.M.B 1007, Unwana, Ebonyi State, Nigeria; bagisuleiman@yahoo.com; 4 Department of Physiology, Faculty of Basic Medical Sciences, College of Medical Sciences, University of Calabar, Calabar, P.M.B. 1115, Calabar, Cross River State, Nigeria; victor2nna@gmail.com; 5 Unit of Integrative Medicine, School of Medical Sciences, Universiti Sains Malaysia, Kubang Kerian 16150, Kelantan, Malaysia

**Keywords:** bee bread, obesity, hyperlipidemia, vasorelaxation, eNOS/NO/cGMP pathway

## Abstract

Obesity and hyperlipidemia are major risk factors for developing vascular diseases. Bee bread (BB) has been reported to exhibit some biological actions, including anti-obesity and anti-hyperlipidemic. This study aims to investigate whether bee bread can ameliorate vascular inflammation and impaired vasorelaxation activity through eNOS/NO/cGMP pathway in obese rats. Forty male Sprague-Dawley rats were randomly divided into four groups (*n* = 10/group), namely: control (normal group), obese rats (OB group), obese rats treated with bee bread (0.5 g/kg/day, OB/BB group) and obese rats treated with orlistat (10 mg/kg/day, OB/OR group). The latter three groups were given a high-fat diet (HFD) for 6 weeks to induced obesity before being administered with their respective treatments for another 6 weeks. After 12 weeks of the total experimental period, rats in the OB group demonstrated significantly higher Lee obesity index, lipid profile (total cholesterol, triglyceride, low-density lipoprotein), aortic proinflammatory markers (tumor necrosis factor-α, nuclear factor-κβ), aortic structural damage and impairment in vasorelaxation response to acetylcholine (ACh). Bee bread significantly ameliorated the obesity-induced vascular damage manifested by improvements in the lipid profile, aortic inflammatory markers, and the impaired vasorelaxation activity by significantly enhancing nitric oxide release, promoting endothelial nitric oxide synthase (eNOS) and cyclic guanosine monophosphate (cGMP) immunoexpression. These findings suggest that the administration of bee bread ameliorates the impaired vasorelaxation response to ACh by improving eNOS/NO/cGMP-signaling pathway in obese rats, suggesting its vascular therapeutic role.

## 1. Introduction

Obesity has contributed to a major burden of disease in developing countries worldwide [1]. Obese individuals are predisposed to vascular diseases, particularly coronary and cerebellar artery diseases, affecting their mobility and mortality as the diseases progress [2]. A dramatic rise in the number of cardiovascular deaths has been projected in the year 2030 [3], and obesity accounted for 54.8% among the risk factors associated with cardiovascular disease (CVD) following hypercholesterolemia (51.5%), hypertension (39.3%), smoking (16.3%), diabetes mellitus (7.8%) and alcohol consumption (1.4%) [4].

Higher circulating free fatty acids contributed by excessive fatty diet trigger the adipose tissue and systemic inflammation, which indicates a crucial interplay between obesity and hyperlipidemia [5]. In the presence of high circulatory lipids, tumor necrosis factor (TNF)-α can activate nuclear factor (NF)-κβ. The acute increase in the level of NF-κβ, therefore, increases the expression of NF-κβ-derived proinflammatory markers. These include adhesion molecules and chemokine inflammatory mediators that are capable of triggering monocyte binding and infiltration into the sub-endothelial region, as well as inducing smooth muscle migration and proliferation, hence, accelerating developing atherosclerosis by causing vascular structural and functional damages [6]. The increase in TNF-α/NF-κβ activation was also seen in the aorta of rats fed with a high-fat diet (HFD) and 10% fructose-induced dyslipidemia for 12 weeks [7].

Studies have shown that obesity-related vascular damage is also linked to a low level of nitric oxide (NO) formation, which is primarily generated by the endothelial layer, the absence of which is described as endothelial dysfunction [8]. In normal and healthy individuals, NO is primarily generated by endothelium nitric oxide synthase (eNOS) in the vascular endothelial layer and plays a crucial role in the regulation of vascular tone and blood flow, allowing for vasorelaxation [9]. NO production might be lost by the destruction of cofactors that are involved in their generation and is regulated by the presence of platelet-derived factors, shear stress, acetylcholine (ACh) and cytokines [10].

There are several pathways, which affect vasorelaxation. However, the most promising therapeutic path that modulates NO is via targeting eNOS/NO/cGMP pathway. NO can stimulate the production of cyclic 3′-5′ guanosine monophosphate (cGMP) signaling through the activation of soluble guanylate cyclase (sGC) to induce smooth muscle relaxation through actin-myosin light chain [11]. The imbalance of functionality and availability of eNOS, which acts through sGC and cGMP, also further reduces functional NO and subsequently impairs the vasorelaxation response [12]. Previous studies that used obese mice [13] and rat [14] models with reduced NO and cGMP levels demonstrated vasorelaxation failure. Therefore, the eNOS/NO/cGMP-signaling pathway plays a critical role in vascular dysfunction in obesity-related vascular damage.

Nowadays, many bee products have been recognized as supplements for humans, as they are rich in nutrients and antioxidants. Propolis, pollen and royal jelly have been reported to produce endothelium-dependent vasorelaxation of arteries in L-NAME-induced hypertensive rats [15,16]. Bee bread is one of the major bee products apart from honey, propolis and royal jelly, and it is commonly used to maintain general health by traditional users in Malaysia. It is made from a mixture of honey, bee pollen, and bee saliva [17]. The bees collect and store them in a beehive, and the bee bread takes about two weeks to be fermented, which gives higher nutritive value and acidity compared to other bee’s products [18]. Bee bread has been shown to possess biological functions such as antimicrobial [19], antitumor [20], and hepatoprotective [21] properties. It has been shown to possess high antioxidant activity due to the presence of active phenolic compounds, such as caffeic acids, ferulic acids, kaempferol, apigenin, and isorhamnetin [22]. Bee bread has a favorable effect on lipid profiling as it demonstrates a reduction in LDL level, and its role as an anti-obesity product was clearly demonstrated in our previous study where bee bread reduced Lee obesity index and the progression of atherosclerosis [22]. However, to date, no study has been reported on the therapeutic role of bee bread, particularly its effect on vascular inflammation in obese rats, and the mechanism involved for its vasorelaxant activity is far from clear. Therefore, this study was designed to determine the ameliorative effect of bee bread on vascular inflammation and impaired vasorelaxation activity in obese male rats, emphasizing eNOS/NO/cGMP-signaling pathway to meet the criteria for the probable vascular therapeutic role.

## 2. Results

### 2.1. Effect of Bee Bread on Lee Obesity Index, Lipid Profile and Atherogenic Ratios in Obese Rats

As shown in Table 1, the OB group had a significant increase in Lee obesity index compared to the normal group. Treatment with bee bread and orlistat significantly reduced the Lee obesity index in the OB/BB and OB/OR groups compared to the OB group. Biochemical analysis of lipid profile was performed to find the changes in lipid levels. The OB group had significantly higher levels of TC, TG and LDL compared to the normal group. Treatment with bee bread significantly reduced these levels, which was comparable to the orlistat group, except for HDL level, which was found to be significantly increased in OB/OR group in respect to the OB group. OB group had significant increases in AI, CRI-1, and CRI-11 by 79.58%, 50.13% and 72.96%, respectively, compared to the normal group. Treatment with bee bread significantly reduced AI, CRI-1 and CRI-11 by 25%, 27.71%, and 28.20%, respectively, compared to the OB group. Meanwhile, treatment with orlistat significantly reduced AI, CRI-1 and CRI-11 by 41.27%, 38.01%, and 33.13%, respectively, compared to the OB group.

### 2.2. Effect of Bee Bread on Aortic Inflammatory Markers in Obese Rats

**Figure 1:**
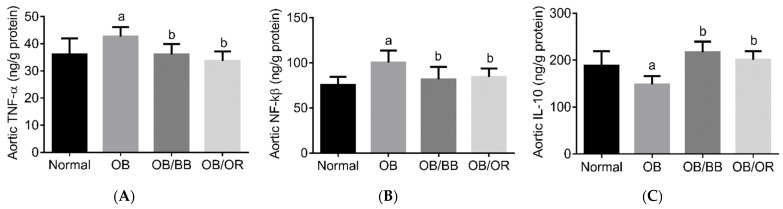
Protein levels of pro- and anti-inflammatory markers (**A**) TNF-α, (**B**) NF-kβ and (**C**) IL-10 in the aorta of all experimental groups. All results are expressed as mean and standard deviation, *n* = 10/group. ^a^ *p* < 0.05 compared to normal group, ^b^ *p* < 0.05 compared to the OB group. All data were analyzed using one-way ANOVA followed by Tukey’s post hoc test. OB: obese, OB/BB: obese and bee bread 0.5 g/kg/day, OB/OR: obese and orlistat 10 mg/kg/day, IL-10: interleukin-10, TNF-α, tumor necrosis factor-alpha, NF-kβ: nuclear factor-kappa β.

Vascular inflammation is an important step in developing vascular damage, which progresses to atherosclerosis. We determined the levels of aortic proinflammatory markers, namely: TNF-α and NF-κβ, as well as the level of the aortic anti-inflammatory marker, IL-10. As indicated in Figure 1, rats in the OB group had a significant increase in the levels of aortic TNF-α and NF-κβ compared to the normal group. Treatment with bee bread and orlistat had significantly abolished the increases of these vascular inflammatory markers compared to the OB group (Figure 1A,B). On the other hand, the level of IL-10 was found to be significantly decreased in the OB group compared to the normal group. Bee bread and orlistat alleviated the vascular inflammation by significantly increasing the aortic IL-10 level compared to the OB group (Figure 1C).

### 2.3. Effect of Bee Bread on Endothelium-Dependent Vascular Reactivity in Obese Rats

**Figure 2:**
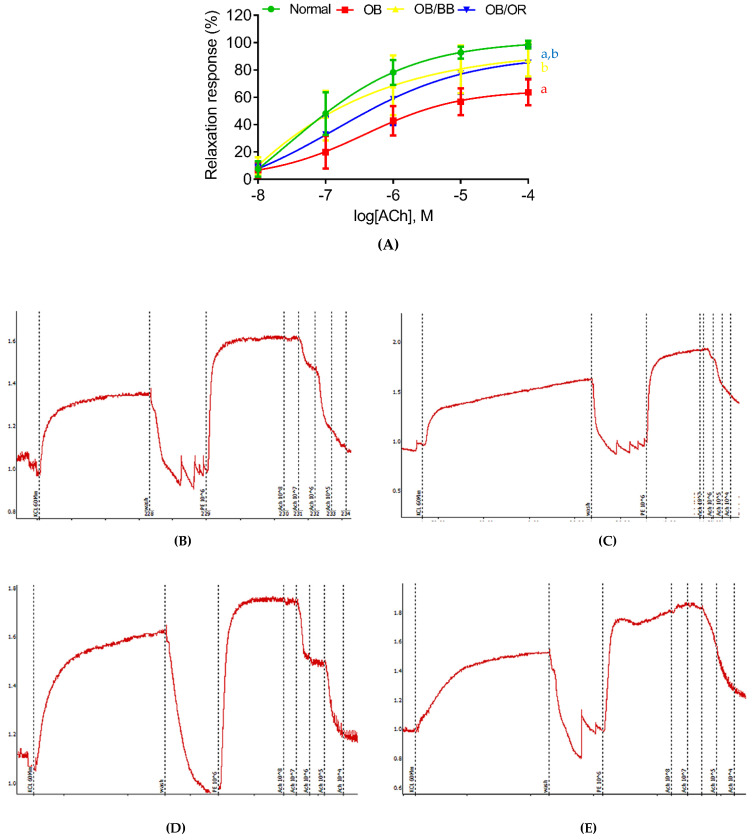
The vasorelaxant effect of bee bread in endothelium-intact thoracic aortic rings precontracted with phenylephrine (10^−6^ M). Fitted cumulative acetylcholine (ACh) dose–response curve is shown in (**A**), and schematic graphs of ACh-induced vasorelaxation activity from (**B**) normal, (**C**) OB, (**D**) OB/BB and (**E**) OB/OR groups are demonstrated. All values are presented as mean and standard deviation (**A**), *n* = 8/group. Data were analyzed using nonlinear regression. ^a^ *p* < 0.05 compared to normal group, ^b^ *p* < 0.05 compared to the OB group. OB: obese, OB/BB: obese and bee bread 0.5 g/kg/day, OB/OR: obese and orlistat 10 mg/kg/day.

Vascular reactivity was performed to determine vascular endothelial responses towards ACh. In normal conditions, ACh acts as an endothelium-dependent vasodilator by inducing relaxation in response to PE-induced vasoconstriction. As shown in Table 2, the endothelium-dependent vasorelaxation induced by ACh in the OB group (E_max_ 65.71%, pE_50_ 6.28) was significantly impaired compared to the normal group (E_max_ 98.43%, pE_50_ 6.77). Bee bread treatment ameliorated these impairments by increasing the endothelium-dependent vasorelaxation response in a dose-dependent manner, with E_max_ of 87.50% yielding a pE_50_ value of 6.77. A similar finding also demonstrated by the OB/OR group, which had a significantly higher E_max_ 82.91% value compared to the OB group and significantly lower value compared to the normal group, without a significant change in pE_50_ value. Bee bread and orlistat had no significant effects on the contraction induced by KCl and PE. Figure 2 demonstrated the percentage of relaxation response (Figure 2A) and schematic graphs (Figure 2B–E) of endothelium-intact thoracic aortic rings precontracted with increasing concentration of PE from all groups. As the concentration of PE increased, the relaxation responses of the thoracic aorta of the OB group were markedly lowered compared to the normal group. Meanwhile, the relaxation responses were markedly increased in the thoracic aorta of OB/BB and OB/OR groups compared to the OB group.

### 2.4. Effect of Bee Bread on Immunoexpression of eNOS and cGMP, and NO Level in the Aorta of Obese Rats

**Figure 3:**
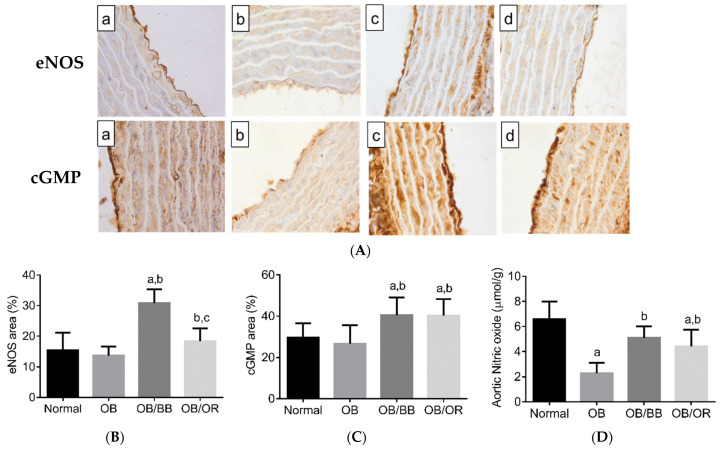
Representative photomicrographs from positive immunohistochemical staining of (**A,B**) eNOS and (**A,C**) cGMP area (brown staining, magnification 400×), and (**D**) aortic NO level in the aorta of (**a**) normal (**b**) OB (**c**) OB/BB and (**d**) OB/OR groups, *n* = 6/group. The quantitative positive staining area of eNOS and cGMP are shown in (**B**) and (**C**), respectively and expressed in percentage value. All data are presented as mean and standard deviation. ^a^ *p* < 0.05 compared to normal group, ^b^ *p* < 0.05 compared to the OB group, ^c^ *p* < 0.05 compared to the OB/BB group. All data were analyzed using one-way ANOVA followed by Tukey’s post hoc test. OB: obese, OB/BB: obese and bee bread 0.5 g/kg/day, OB/OR: obese and orlistat 10 mg/kg/day, eNOS: endothelial nitric oxide synthase, cGMP: cyclic guanosine monophosphate, NO: nitric oxide.

To further confirm the participation of eNOS in endothelium-dependent vasorelaxation, we determined the levels of eNOS and cGMP proteins and NO levels in the aorta. As shown in Figure 3, the mean area for positive immune cells of eNOS (Figure 3A,C) and cGMP (Figure 3B,D) proteins and the level of aortic NO (Figure 3E) were decreased in the OB group when compared to the normal group. In contrast, the levels of eNOS and cGMP proteins and NO levels increased significantly in OB/BB and OB/OR groups when compared to the OB group.

### 2.5. Effect of Bee Bread on Morphology of Aorta in Obese Rats

**Figure 4:**
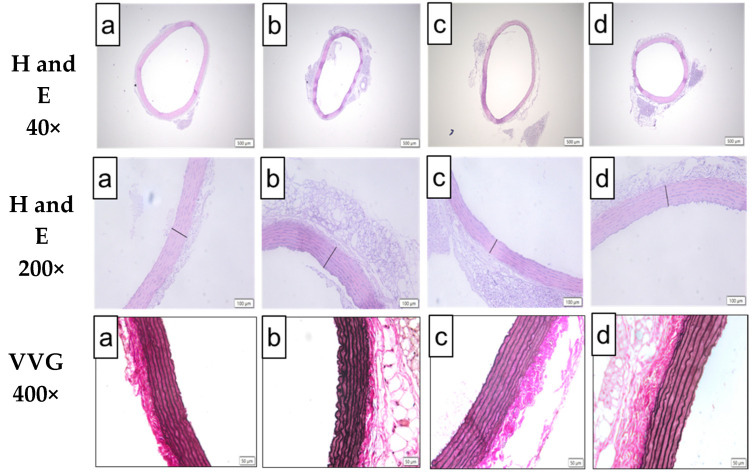
Representative photographs of aortic arch sections stained with H&E (magnification = 40× and 200×, scale bar = 500 µm and 100 µm) and VVG (magnification 400×, scale bar = 50µm) from (**a**) normal, (**b**) OB, (**c**) OB/BB and (**d**) OB/OR groups. H&E staining showed the presence of normal aorta lining in the normal group, while a thicker aorta was observed in the OB group compared to the normal group. The thickness of the aorta was also markedly reduced in OB/BB and OB/OR groups compared to the OB group. VVG staining showed the presence of wavy, irregular and tortuous elastic lamella in the OB group and the internal lamella lining was homogenously improved in (**c**) and (**d**) groups compared to (**b**) OB: obese, OB/BB: obese, and bee bread 0.5 g/kg/day, OB/OR: obese and orlistat 10 mg/kg/day, H&E: hematoxylin and eosin, VVG: Van Verhoef Gielson.

**Figure 5:**
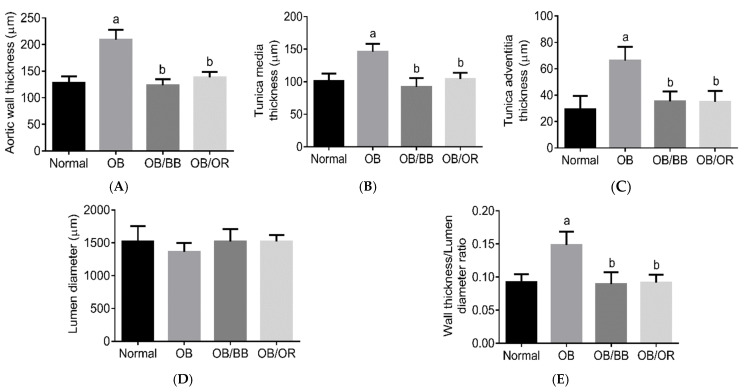
Morphometric analysis of H&E stained aorta from each experimental group. Values are mean and standard deviation, *n* = 10/group. Thickness of (**A**) aortic wall, (**B**) tunica media, and (**C**) tunica adventitia, (**D**) lumen diameter and (**E**) ratio of wall thickness per lumen diameter of the thoracic aorta. OB: obese, OB/BB: obese and bee bread 0.5 g/kg/day, OB/OR: obese and orlistat 10 mg/kg/day. ^a^ *p* < 0.05 compared to normal group, ^b^ *p* < 0.05 compared to the OB group. All data were analyzed using one-way ANOVA followed by Tukey’s post hoc test.

Figure 4 demonstrates the representative histological findings of H&E (Figure 4A,B) and VVG (Figure 4C)-the stained aorta and the morphometric analysis from H&E-stained aorta from each experimental group (Figure 5). Microscopic changes of the H&E-stained aorta revealed the presence of a significantly thickened aortic wall in the OB group, which was associated with significantly thicker tunica intima and adventitia layers compared to the normal group. These effects were also found to be associated with a significant increase in wall thickness per lumen ratio in the OB group compared to the normal group. Treatment with BB and orlistat significantly restored all these changes with an observation of a significant decrease in the aortic wall thickness, including tunica intima and adventitia, compared to the OB group. The VVG-stained aorta of the normal group demonstrated the presence of linear and homogenous lining of the elastic lamella in the tunica media. Meanwhile, the VVG-stained aorta of the OB group showed architecture characteristic of irregularity and tortuous elastic lamellae in the tunica media, associated with the presence of rough endothelial surface compared to the normal group, and the lining of elastic lamellae seems to be markedly improved in the aortas of the OB/BB and OB/OR groups when compared to the OB group.

### 2.6. Mineral Composition of Bee Bread

The bee bread used in the present study showed a broad spectrum of mineral composition. Our result demonstrates that the major electrolytes, which are abundantly present in bee bread, are potassium (7323.04 mg/kg), magnesium (1530.87 mg/kg) and calcium (1108.48 mg/kg), followed by sodium (252.73 mg/kg). In addition, it also possesses trace minerals, namely: iron (56.58 mg/kg), zinc (42.36 mg/kg), copper (11.05 mg/kg) and selenium (0.13 mg/kg) (Table 3).

## 3. Discussion

Our study demonstrated that bee bread treatment at 0.5 g/kg/day for 6 weeks exerted a therapeutic effect on obesity-induced impairment in ACh-mediated vasorelaxation response, in addition to ameliorating aortic inflammation and structural changes, obesity index, hyperlipidemia and atherogenic ratios. Our study also has pointed to the mechanistic role of aortic eNOS/NO/cGMP-signaling pathway in obesity-induced impairment in ACh-mediated vasorelaxation response.

Hyperlipidemia is one of the features of obesity, leading to the development and progression of vascular dysfunction that could be attributed to atherosclerosis [23]. Excessive intake of diets containing high dietary fat contributes to fat accumulation in the body and results in obesity. We used the Lee obesity index as a tool to determine obesity, where a value higher than 315 was categorized as obese [24]. The present findings showed that chronic intake of HFD for 12 weeks caused a large accumulation of body fat mass, as evident in a significant increase in Lee obesity index demonstrated in the OB group. The increase in Lee obesity index contributed to the increased fat accumulation in the body. In the present study, the continuous HFD ingestion may have resulted in enormous TG production and storage in the adipose tissue, increased sizes and number of adipocytes, and increased adiposity. This was supported by the higher atherogenic ratios (AI, CRI-1 and CRI-11), representing a higher risk of developing atherosclerosis [25]. The weight-reducing and cholesterol-lowering effects of bee bread observed in the present study agree with our previous study [22]. The significant reduction in lipid fractions found in the OB/BB group may be related to the presence of active polyphenols, such as caffeic and ferulic acids, which have lipid-lowering effects, as demonstrated in previous studies [22,26,27]. Moreover, the mechanism by which bee bread reduced the serum non-HDL lipids concentration may be due to the action of saponin in the bee bread because saponin has been reported to bind to dietary fat constituents, and in turn, increase fat excretion in feces [28]. The significant reductions in TC, TG and LDL levels, with an increase in HDL level in the OB/OR group, are consistent with a previous study that administered 30 mg/kg orlistat, a potent gastropancreatic lipase inhibitor, for 8 weeks in an obese rat model [29]. Orlistat has been reported to increase the paraoxonase activity of HDL particles, hence, increase its antioxidant activity by preventing the oxidation of lipid peroxidation products, particularly the pro-atherogenic LDL-cholesterol [30]. Therefore, both bee bread and orlistat could mitigate atherosclerosis progression by targeting hyperlipidemia.

The initiation of vascular dysfunction is not only caused by increased lipid concentration but also due to a cascade of inflammatory processes, which is a crucial factor for atherosclerosis progression manifested by increased cytokine release [31]. TNF-α is a potent activator for NF-κβ, which is crucial for the generation of other proinflammatory markers, such as the leukocyte adhesion molecules and chemokines, which can further initiate vascular dysfunction [6]. In the present study, we quantified proinflammatory TNF-α and NF-κβ in the aorta, which are important mediators of atherogenesis, together with the level of the anti-inflammatory cytokine, IL-10. In normal physiological conditions, NF-kβ is sequestered by Ikβα, while increased TNF-α causes the degradation of Ikβα, hence allowing the translocation of NF-κβ into the nucleus where it initiates the expression of target genes encoding for leukocyte adhesion molecule and chemokine release [32]. The presence of inflammation, as shown by the significantly elevated levels of aortic TNF-α and NF-κβ and decreased aortic IL-10 level in the OB group, is consistent with the finding in the testis of the obese rat model [33]. Meanwhile, both bee bread and orlistat significantly suppressed the increased aortic TNF-α and NF-κβ levels, indicating their anti-inflammatory actions, which are consistent with previous findings in the kidney of obese rats [34]. The improvement of these inflammatory markers in the OB/BB group may be related to the inhibition of NF-κβ activity and enhancing the activity of IL-10 by the flavonoids present in the bee bread, such as isorhamnetin kaempferol and quercetin [35]. Our previous study demonstrated that orlistat exerted anti-inflammatory properties by significantly suppressing the increase in expression of NF-κβ derived pro-atherogenic markers of adhesion molecules, such as vascular cell adhesion molecule (VCAM-1) and intracellular cell adhesion molecule (ICAM-1) [36]. Therefore, NF-κB becomes an interesting therapeutic target to combat the progression of atherosclerosis. However, it is unclear how bee bread and orlistat could ablate the TNF-α/NF-κβ signaling and thus require a detailed study to determine their exact molecular mechanisms of action.

The endothelium is a single-cell receptor-rich lining of the inner layer of the intima that plays a critical role in the regulation of vascular tone, and thus, is a primary target for vasodilatory action [37]. Endothelial damage could contribute to developing atherosclerosis and loss of endothelial-dependent vasorelaxation [38]. Our findings revealed an impairment in the dose-dependent ACh-mediated vasorelaxation of aortic rings in the OB group (E_max_ 65.71%, pE_50_ 6.28) compared to the normal group (E_max_ 98.43%, pE_50_ 6.77), indicating the failure of endothelial function to maintain the vascular tone. Meanwhile, the endothelial-dependent vasorelaxation of the aortic rings in the OB/BB and OB/OR groups showed significant improvements, in a concentration-dependent manner, compared to the OB group, which may indicate the improvement of endothelial function. In the present study, bee bread has demonstrated its ameliorative effect on the impairment of ACh-induced endothelium-dependent relaxation that is comparable to orlistat, without any changes in maximal relaxation towards KCl and PE. Several mechanisms may account for developing endothelial dysfunction in obesity. These include the presence of pro-atherogenic LDL cholesterol in the subendothelial region that triggers an aortic inflammatory reaction by activating the TNF-α/NF-κβ pathway, hence accelerates the progression of atherosclerosis [39]. The ongoing inflammatory process involves the migration and proliferation of the vascular smooth muscle cell [40] that subsequently increases the interlaminar space, extracellular matrix content and further destroys the normal alignment of elastic fibers of tunica media as demonstrated in VVG-stained aorta in the OB group. Therefore, these changes could then affect the structural integrity of the vessel, which subsequently impairs the vasodilatory response. On the other hand, the changes in the lumen diameter of the vessels can also influence the tissue perfusion and debilitate the response of vessels towards vasodilators and vasoconstrictors [41]. Therefore, we speculated that the increase in the atherogenic LDL cholesterol, aortic TNF-α/NF-κβ activation, thicken aortic wall, and distorted elastic lamellae in the tunica media found in the OB group might be the factors that contributed to the impairment of the structural integrity of the aorta and therefore, impaired its endothelial-dependent vasorelaxation response. Treatment with bee bread and orlistat, however, ameliorated these morphological changes. The restored alignment of the elastic lamellae in the treatment groups, when compared to the OB group, might imply the ameliorative effects of bee bread and orlistat, hence improved the endothelial-dependent vasorelaxation response in our obesity model. This could be due to its hypolipidemic and anti-inflammatory effects, which need further study to determine the exact molecular mechanism of action.

Studies have shown that the relaxation responses of the vessels are primarily attributed to the presence of NO, a potent vasodilator, which is primarily generated by eNOS in the endothelium [39,42]. The generated NO diffuses from the endothelial layer to the smooth muscle, where it activates soluble guanylyl cyclase (sGC) and converts guanosine triphosphate (GTP) to cGMP, leading to smooth muscle relaxation [43]. Hence, the lower level of NO and immuno-expressions of eNOS and cGMP proteins which are similar to previous studies [44,45], could explain the observed impairment of vasorelaxation response found in the OB group. However, bee bread and orlistat treatments ameliorated these changes. The level of NO is increased following the phosphorylation of eNOS, which results in its functional activity. However, in a hyperlipidemic state, the level of phosphorylated eNOS is reduced by the action of oxidized pro-atherogenic LDL that inhibits the eNOS coupling and activation [46]. Therefore, it is plausible to suggest that bee bread and orlistat improved the NO level by enhancing eNOS expression, which in turn improved the vasorelaxation response to ACh in the present study. Studies have found that several natural products could also increase eNOS activity and, therefore, increase the vasorelaxant responses [47,48]. For example, white tuber extract has been reported to improve relaxation response to ACh and increase eNOS expression via improvement of NO/cGMP-signaling pathway in aortic tissue of rats fed with HFD [14]. The enhancement of eNOS expression in the OB/BB group could also be attributed to the presence of active compounds in bee bread, which stimulates the phosphorylation of eNOS, thereby increase its activation and production of functional NO.

**Figure 6:**
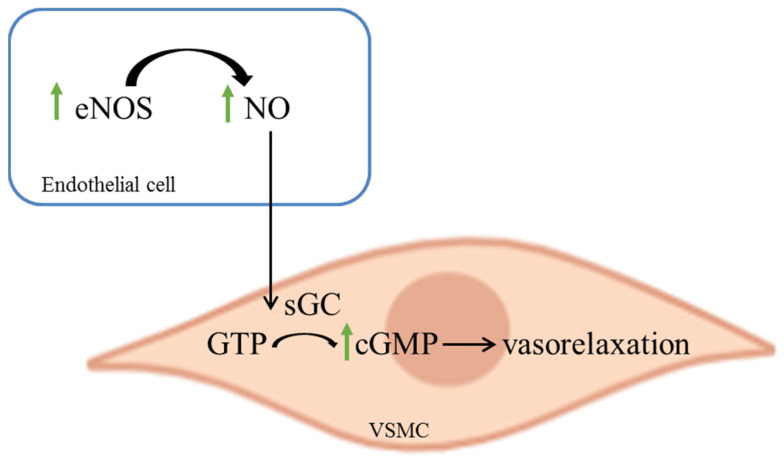
Schematic diagram of the mechanism of bee bread (green arrowhead) induced vasorelaxation in the isolated aorta of obese rats. eNOS: endothelial nitric oxide synthase, NO: nitric oxide, GTP: guanosine triphosphate, cGMP: cyclic guanosine monophosphate, VSMC: vascular smooth muscle cell.

The bee bread that was used in our study contains a broad spectrum of trace minerals (iron, zinc, copper and selenium) and electrolytes (calcium, potassium, magnesium and sodium). These minerals are essential as they act as cofactors and mediators for physiological and biochemical functions in the body. Hence, we speculate that the presence of broad-spectrum minerals in the bee bread may also contribute to the improvement of vascular reactivity in this obesity model. For instance, both copper and zinc are involved as ligands in the superoxide dismutase (SOD1) system, which catalyzes the dismutation of highly toxic superoxide radicals into oxygen and hydrogen peroxide [49]. The improvement of the structure and function of the aorta in rats treated with bee bread in our study may also be related to the presence of this oxidoreductase enzyme via its action in reducing the pro-atherogenic oxidized LDL. Magnesium has a regulatory role on blood pressure since it can improve the vascular tone and mediate vascular smooth muscle contraction. Studies have shown that maintaining the magnesium level in the body can improve vascular reactivity and impede endothelial dysfunction, as well as prevent the associated cardiovascular health-related problems, such as obesity, hypertension and stroke [50]. Taken together, we hypothesize that bee bread treatment ameliorates the impaired ACh-mediated vasorelaxation in the aorta of obese rats via eNOs/NO/cGMP pathway, which was comparable with orlistat treatment (Figure 6).

## 4. Materials and Methods

### 4.1. Chemical and Drugs

Sodium chloride, potassium chloride, calcium chloride dehydrate, magnesium sulfate anhydrous and potassium dihydrogen phosphate were purchased from Merck (Emsure^®^ Merck KGaA, Darmstadt, Germany), while sodium hydrogen carbonate was purchased from Riedel-de Haën (Seelze, Germany). D (+)-Glucose monohydrate was obtained from HmbG^®^ Chemicals (Hamburg, Germany), Acetylcholine chloride from Nacalai Tesque, Inc. (Kyoto, Japan) and (R)-Phenylephrine Hydrochloride from TCI Development Co., Ltd. (Shanghai, China). All other reagents and solvents were analytical grade.

### 4.2. Preparation of Bee Bread

Bee bread was purchased from a local beekeeper farm (Mentari Technobee PLT), Kelantan, Malaysia. Fresh bee bread was dried using a food dehydrator at 35 °C until a constant weight was achieved, followed by crushing in an industrial blender into powder form. The powder was kept at −20 °C until further use.

### 4.3. Animals and Diet

Forty male *Sprague-Dawley* rats (weighing 180–230 g, aged 8–10 weeks old) were purchased from the Animal Research and Service Centre, Universiti Sains Malaysia. They were housed in individual cages with a controlled temperature at 22 ± 2 °C, relative humidity 60–70%, and 12 h light/dark cycle. All rats were given a normal chow diet and water ad libitum during one week of the acclimatization period. The rats were fed either a normal diet (Altromin Spezialfutter GmbH and Co. KG, Lage, Germany) or a high-fat diet (HFD), which was prepared according to our previous study [22]. The normal diet comprised of 64% carbohydrate, 24% protein, and 12% fat, which contributes to 31.8 kcal/g, while the HFD comprised of 46% carbohydrate, 12% protein and 31% fat, which contribute to 51.65 kcal/g.

### 4.4. Experimental Design

The study is divided into two phases. In phase 1, the rats were randomly divided into two groups, i.e., a normal group (*n* = 10) and a high-fat diet (HFD) group (*n* = 30). The rats in the normal group received a normal chow diet, while rats in the HFD group were administered with HFD for 6 weeks to induce obesity characterized by a high Lee obesity index of more than 315. In phase 2, which also lasted for 6 weeks, the normal group continued to receive a normal chow diet. Obese rats from phase 1 were subjected to treatment phase with continuous administration of HFD either with distilled water (OB group), bee bread at 0.5 g/kg/day (OB/BB group), or orlistat at 10 mg/kg/day (OB/OR group) via oral gavage. The corrected amount of bee bread and orlistat were based on the respective weight of the rats. Both bee bread and orlistat were separately suspended in 1 mL of distilled water before being administered to the rats daily. Bodyweight and food intake were determined once a week and every day, respectively, during the entire experimental period. All animal procedures were handled following the National Institute of Health Guide for the Care and Use of Laboratory Animals, and USM animal ethics approved the study (USM/IACUC/2018/(113)(933)). All efforts were made to minimize animal suffering. At the end of the experimental period, all rats were sacrificed after being euthanized with ketamine 90 mg/kg and xylazine 5 mg/kg. Central blood was collected from the posterior vena cava for serum analysis. The thoracic aorta was immediately excised for analysis on vascular reactivity. The aortic arch was homogenized with 10% 0.1 M phosphate-buffered saline (*w/v*) for biochemical analysis, and the abdominal aorta was divided into sections for histological analysis.

### 4.5. Measurement of Obesity Parameters

Lee obesity index was determined following a previously described method, and a value >315 was considered obese [24]. The Lee index was calculated by dividing the cube root of the bodyweight (gram) by the naso-anal length (cm). The obtained result was multiplied by 1000.

### 4.6. Measurement of Lipid Profile and Atherogenic Ratios

The collected blood was allowed to stand for 2 h at room temperature, and the serum was separated by centrifugation at 4000× *g* for 15 min. Total cholesterol (TC) and triglyceride (TG) were measured by an enzymatic-colorimetric method using Architect c total cholesterol and triglyceride kits (ARCHITECT c kit, Abbott, IL, USA). The low-density lipoprotein (LDL) was calculated using the equation: LDL (mg/dL) = TC—HDL—(TG/5) [51]. High-density lipoprotein (HDL) was determined by Biosino Direct HDL-Cholesterol reagent kit (Biosino Bio-Technology and Science Inc., Beijing, China). Atherogenic ratios are predictive factors for atherosclerosis and were calculated as follows:

Atherogenic index = (TC-HDL)/HDL [52];
Castelli’s risk index (CRI-I) = TC/HDL [53];
Castelli’s risk index (CRI-II) = LDL/HDL [25].

### 4.7. Determination of Aortic Inflammatory Markers

The aortic homogenate was used to quantify proinflammatory (TNF-α and NF-κβ) and anti-inflammatory cytokine (interleukin-10, IL-10), which may contribute to the presence of vascular damage by adopting rat enzyme-linked immunosorbent assay (ELISA) supplied by Qayee-Bio (Shanghai, China) according to the manufacturer’s protocol. The absorbance value was read at 450 nm wavelength.

### 4.8. Determination of Aortic NO Level

NO was quantified in aortic homogenate using a NO assay detection kit (Elabscience, Wuhan, China) according to the manufacturer’s instructions. Since NO has a short half-life, the kit measures the levels of NO metabolites, i.e., nitrate and nitrite. Following a color change at room temperature, the absorbance value was determined using a microplate reader at 550 nm wavelength. Sodium nitrite was used as a standard. Total NO for each sample was normalized with protein concentration and expressed as µmol/g protein.

### 4.9. Preparation of Aorta and Measurement of Vascular Reactivity

The thoracic aorta was dissected out and transferred into a petri-dish containing ice-cold, pre-carbonated (95% O_2_, 5% CO_2_) Krebs–Henseleit solution (118 mM NaCl, 4.7 mM KCI, 1.18 mM MgSO_4_·7H_2_O, 1.2 mM KH_2_PO_4_, 2.0 mM CaCl_2_·2H_2_O, 25 mM NaHCO_3_ and 5.5 mM D-glucose, pH 7.4) [54]. The aorta was carefully handled and removed from adhesive fat and connective tissue before being divided into three segmental rings, approximately 5 mm in size. Individual rings of the aorta were inserted with two stainless steel wires into the aortic lumen and bathed in a prewarmed (37 °C) tissue bath chamber containing 10 mL of Krebs–Henseleit solution and connected to an Automatic organ bath (PanLab. LE01026, Panlab, MA, USA) through a force transducer. The tissue bath containing Krebs–Henseleit solution was continuously aerated with 95% O_2_ and 5% CO_2_ to maintain the pH of 7.4. Isometric tension of 1 g was applied to the aorta for 45 min during the equilibrium phase. The tension was adjusted as required, and the Krebs–Henseleit solution was changed every 20 min. After equilibrium, the aortic ring was challenged with 60 mM KCl to obtain reference contraction. Thereafter, the aortic rings were precontracted with PE 10^6^ M followed by cumulative concentrations of ACh, after which the maximum contraction of PE 10^6^ M had reached its level. The aortic relaxation by cumulative addition of ACh was performed with intact endothelium to investigate the endothelium-dependent vasorelaxation. All changes in isometric tension were detected with a force transducer and recorded using LabChart^®^ Reader. The vasorelaxation response was calculated in percentage of the contraction produced by PE as E_max_. Maximal contractions of aortic rings toward KCl and PE were also expressed in percentage.

### 4.10. Immunohistochemistry

The paraffin-embedded aortic segment was sectioned at 5 µm thickness onto a silane-coated slide (microslides, Muto Pure Chemicals Co., Ltd., Tokyo, Japan). After being placed on a hot plate at 60 °C for 10 min, the slides were hydrated with different concentrations of ethanol. Antigen retrieval was performed by placing the slides into a pressure cooker filled with a tris-EDTA buffer with 0.05% tween-20 (pH 9.0) for 3 min. The sections were immersed in 3% hydrogen peroxide for 5 min to block the endogenous peroxidase before incubating with polyclonal primary antibodies for cGMP (1:100, Frieldwelt, USA) and eNOS (1:20, CloudClone, China) in a humidified chamber overnight. Afterward, the sections were incubated with Dako Envision^TM^+ System/HRP-labeled polymer (Dako North America, Inc., Carpinteria, CA, USA) containing goat anti-rabbit secondary antibody for 1 h. Specific labeling of the antigen was visualized by diaminobenzidine (Dako Liquid DAB+ Substrate Chromogen System, Dako North America, Inc.). The slides were counterstained with hematoxylin solution, and the positive immunostaining areas were examined under a light microscope (Leica, Wetzlar, Germany). Photographs were taken in five non-overlapping fields in each paraffin block for each rat (*n* = 6/group) using 100× objective. The mean of the stained area of the five fields was used as a representative for an individual rat and quantified using ImageJ software (ImageJ, NIH, Bethesda, MD, USA).

### 4.11. Aorta histological Analysis

Isolated aorta from each group was fixed in 10% formalin (*v/v*) for 24–48 h. The tissue was excised and processed using an automated tissue processor (Leica, Wetzlar, Germany) and subsequently embedded in Paraplast (Sigma-Aldrich, St. Louis, MO, USA). Afterward, the tissue was sectioned at 5 µm thickness using a microtome (Leica RM2235, Walldorf, Germany) before being stained with hematoxylin and eosin (H&E), and Verhoeff–Van Gieson’s (VVG) method, respectively [37]. H&E-stained aortic sections were used to measure the thicknesses of the aortic wall, tunica media, tunica adventitia (without the perivascular adipose tissue), and lumen diameter. Meanwhile, VVG staining was performed to compare the lining of internal lamella in the tunica media layer. For histomorphometry of the H&E-stained aorta, photographs were taken from five different non-overlapping fields (*n* = 10/group) using a 40 × objective. Each photograph was used for the morphometry analysis using the straight segmented or freehand lines tools of the cell sense system in Image analyzer (Olympus, Tokyo, Japan). The mean value of the five fields was used as a representative for each rat.

### 4.12. Mineral Analysis of Bee Bread

Thirty grams of powdered bee bread was utilized for analysis of the mineral composition. Microwave-assisted extraction was performed using a Milestone microwave laboratory system (MLS-1200 Mega, Gemini BV, Apeldoorn, The Netherlands). The yield stock solution was used for the analysis of mineral composition by using inductively coupled plasma mass spectrometry (ICP-MS) (Elan 9000, PerkinElmer Instrument, Waltham, MA, USA).

### 4.13. Statistical Analysis

All data were analyzed using GraphPad Prism version 6 (GraphPad Software Inc., San Diego, CA, USA). Shapiro–Wilk and D’Agostino–Pearson’s omnibus tests were used to confirm that all data were normally distributed and homogenous, respectively. Then, One-way analysis of variance (ANOVA) followed by Tukey’s post hoc test was used for the determination of significant differences between the experimental groups, and the results were expressed as mean (standard deviation). Differences with a *p* value of < 0.05 were considered statistically significant. Relaxation responses were expressed as a percentage decrease of the maximal PE contraction. The cumulative dose-dependent curve was plotted on the application of sigmoidal curve fitting and nonlinear regression as maximum relaxation (E_max_) and pE_50_ (negative logarithm of the concentration of ACh that was needed to produce 50% of the maximum aortic relaxation response).

## 5. Conclusions

Bee bread treatment at 0.5 g/kg/day for 6 weeks following obesity induction significantly improved the lipid profile, atherogenic ratios, aortic inflammatory markers and the morphological changes of the aorta in rats. These changes cumulatively resulted in the ameliorative effect of bee bread on impaired vasorelaxation response to ACh, which could involve eNOS/NO/cGMP-signaling pathway (Figure 6). Further studies are required to determine its exact molecular mechanism of action, in addition to the possibility of mimicking the actions of statins in inhibiting HMG-CoA reductase. In addition, further study is also suggested to evaluate the possible effects of bee bread and orlistat on these parameters in rats fed with a normal diet.

## Figures and Tables

**Table 1:** Effect of bee bread on Lee obesity index, lipid profile and atherogenic ratios.

Parameters	Normal	OB	OB/BB	OB/OR
Lee obesity index	304.85 (7.20)	332.13 (7.15) ^a^	311.42 (6.94) ^b^	316.70 (7.81) ^b^
TC (mmol/L)	1.61 (0.16)	2.63 (0.65) ^a^	1.99 (0.15) ^b^	2.05 (0.20) ^b^
TG (mmol/L)	0.50 (0.08)	0.92 (0.10) ^a^	0.59 (0.16) ^b^	0.66 (0.14) ^b^
LDL (mmol/L)	0.93 (0.19)	1.65 (0.57) ^a^	1.10 (0.35) ^b^	1.08 (0.24) ^b^
HDL (mmol/L)	0.41 (0.10)	0.40 (0.05)	0.49 (0.10)	0.66 (0.09) ^a,b,c^
Atherogenic index	2.361 (0.57)	4.24 (0.73) ^a^	3.18 (0.52) ^b^	2.49 (0.62) ^b^
Castelli risk index 1	3.75 (0.96)	5.63 (0.33) ^a^	4.07 (0.47) ^b^	3.49 (0.62) ^b^
Castelli risk index 11	2.04 (0.56)	3.53 (0.69) ^a^	2.53 (0.33) ^b^	2.36 (0.51) ^b^

Data are presented as mean (standard deviation), *n* = 10/group. ^a^ *p* < 0.05 compared to normal group, ^b^ *p* < 0.05 compared to the OB group, ^c^ *p* < 0.05 compared to the OB/BB group. All data were analyzed using one-way ANOVA followed by Tukey’s post hoc test. OB: obese, OB/BB: obese and bee bread 0.5 g/kg/day, OB/OR: obese and orlistat 10 mg/kg/day, TC: total cholesterol, TG: triglyceride, LDL: low-density lipoprotein, HDL: high-density lipoprotein.

**Table 2:** Ex vivo aortic isolated tissue bath assay.

Parameters	Normal	OB	OB/BB	OB/OR
E_max_ KCl (g)	0.44 (0.08)	0.47 (0.18)	0.55 (0.13)	0.44 (0.1)
E_max_ PE (g)	0.60 (0.14)	0.66 (0.15)	0.71 (0.13)	0.64 (0.14)
E_max_ ACh (%)	98.43 (3.01)	65.71 (11.14) ^a^	87.50 (12.38) ^b^	82.91 (12.28) ^a,b^
pEC50	6.77 (0.07)	6.28 (0.10) ^a^	6.77 (0.15) ^b^	6.43 (0.12)

Data are presented as mean (standard deviation), *n* = 10/group. ^a^ *p* < 0.05 compared to normal group, ^b^ *p* < 0.05 compared to the OB group. All data were analyzed using one-way ANOVA followed by Tukey’s post hoc test. OB: obese, OB/BB: obese and bee bread 0.5 g/kg/day, OB/OR: obese and orlistat 10 mg/kg/day, E_max_: maximum relaxation, KCl: potassium chloride, PE: phenylephrine, ACh: acetylcholine, pEC50 is the negative logarithm of the concentration of ACh that gives a half-maximal response.

**Table 3:** Mineral and vitamin contents of bee bread.

Minerals	Composition (mg/kg)
Calcium	1108.48
Copper	11.05
Iron	56.58
Magnesium	1530.87
Potassium	7323.04
Selenium	0.13
Sodium	252.73
Zinc	42.36

## Data Availability

The data are presented within the paper. Additional raw data are available on request from the corresponding author.
